# Soil Moisture Retrieval by Integrating TASI-600 Airborne Thermal Data, WorldView 2 Satellite Data and Field Measurements: Petacciato Case Study

**DOI:** 10.3390/s19071515

**Published:** 2019-03-28

**Authors:** Angelo Palombo, Simone Pascucci, Antonio Loperte, Antonio Lettino, Fabio Castaldi, Maria Rita Muolo, Federico Santini

**Affiliations:** 1 Consiglio Nazionale delle Ricerche (CNR), Piazzale Aldo Moro, 7, 00100 Rome, Italy; angelo.palombo@cnr.it; 2 Consiglio Nazionale delle Ricerche—Institute of Methodologies for Environmental Analysis (C.N.R.—IMAA), C.da S.Loja—Zona Industriale, Tito Scalo, 85050 Potenza, Italy; antonio.loperte@cnr.it (A.Lo.); antonio.lettino@cnr.it (A.Le.); federico.santini@cnr.it (F.S.); 3 Georges Lemaître Centre for Earth and Climate Research, Earth and Life Institute, Universitè Catholique de Louvain, Croix du Sud 2,L7.05.16, 1348 Louvain la Neuve, Belgium; fabio.castaldi@uclouvain.be; 4 Servizi di Informazione Territoriale S.r.l., P.zza Papa G.Paolo II, 8/1, 70015 Noci, Italy; mr.muolo@sit-puglia.it

**Keywords:** thermal multispectral data, soil moisture, TASI-600, thermal inertia, data integration

## Abstract

Soil moisture (SM) plays a fundamental role in the terrestrial water cycle and in agriculture, with key applications such as the monitoring of crop growing and hydrogeological management. In this study, a calibration procedure was applied to estimate SM based on the integration of in situ and airborne thermal remote sensing data. To this aim, on April 2018, two airborne campaigns were carried out with the TASI-600 multispectral thermal sensor on the Petacciato (Molise, Italy) area. Simultaneously, soil samples were collected in different agricultural fields of the study area to determine their moisture content and the granulometric composition. A WorldView 2 high-resolution visible-near infrared (VNIR) multispectral satellite image was acquired to calculate the albedo of the study area to be used together with the TASI images for the estimation of the apparent thermal inertia (ATI). Results show a good correlation (R^2^ = 0.62) between the estimated ATI and the SM of the soil samples measured in the laboratory. The proposed methodology has allowed us to obtain a SM map for bare and scarcely vegetated soils in a wide agricultural area in Italy which concerns cyclical hydrogeological instability phenomena.

## 1. Introduction

Soil moisture (SM) is a variable that is influenced by a wide range of processes that occur in the land–atmosphere interface, including water infiltration, water outflow, evaporation, heat and gas exchange, infiltration of solutes, erosion, etc. Different studies have demonstrated that SM is a function of different processes (precipitation, evapotranspiration, etc.) and is influenced by various factors, e.g., topography, land use, and soil texture. SM is highly varied in space and time and across different scales [1,2,3,4,5].

Many authors has highlighted the importance of measuring and monitoring SM and, in particular, the water in the upper 10 cm of soil at different spatial scales [2,3,4,5]. Several satellite missions including SMOS (Soil Moisture and Ocean Salinity, [6]), SMAP (Soil moisture Active and Passive, [7]), ASCAT (Advanced Scatterometer [8]), provide SM estimates at coarse spatial resolution and very high revisit time (up to 1 day). Some practical applications of SM retrieval can be found in the works of [9,10]. However, only recently SM maps up to plot scale with high revisit time (5 to 6 days) have been derived from the integration of Sentinel-1 radar and Sentinel-2 optical imagery [11]. However, despite recent advances in remote sensing [12,13,14,15,16], an accurate estimate with a high spatial resolution of surface water content in soils by remote methods is still a challenging task due to SM spatial–temporal variability and to the scale problems characteristic of this kind of applications [17]. In situ or remotely sensed observations of SM for initialization, update, and validation purposes are not yet available on the scales of most models. Observations are generally confined to short-term field experiments, many of which have highlighted the heterogeneous nature of soils in terms of water content and texture [18].

The surface moisture content and its interaction with soil is highly correlated with the electromagnetic radiation at various wavelengths, from the visible (VNIR) to the thermal infrared (TIR) spectral regions. Many scientists [19,20,21,22,23,24,25,26] have long recognized the effect of SM on the reflectance spectrum. For example, [27] developed a physical model to explain the soil reflectance variations due to moisture change based on their analysis of the reflectance for different soils at various moisture contents. Moreover, different authors have modeled the soil reflectance, as an exponential function of the volumetric SM, e.g., [28] calibrated a normalized SM index suitable for hyperspectral remote sensing data, exploiting the spectral features correlated with SM at 1800 and 2119 nm. Hajj et al. [11] have recently mapped SM over agricultural areas by the synergic use of Sentinel-1 SAR and Sentinel-2 optical images. Their results show that the use of a priori information on the SM condition increases the precision of the SM estimates with an accuracy of approximately 5 vol % (volumetric unit expressed in percent). Wang and Qu [4], in their review article on recent satellite remote sensing applications for surface SM monitoring, argue that accurate estimates of the spatial and temporal variations of SM is critical for numerous environmental studies and SM can be measured using different remote sensing techniques, each with its own strengths and weaknesses. Zhang and Zhu [29] provided a detailed review on the use of optical and thermal remote sensing data for SM assessment, concluding that the integration of VNIR and TIR data can provide accurate information for SM estimation.

Concerning the estimation of surface SM using the TIR spectral range (3.5–14 μm), it is mostly based on surface temperature measurements, or on the use of thermal inertia (TI) [30,31,32]. This because the thermal properties that control the daily range of soil temperature are the soil thermal conductivity and the soil heat capacity; therefore, variations in SM have a strong impact on soil thermal properties being an intrinsic factor of soil surface temperature change [4,33]. Watson [34] first proposed physical analytical equations of TI retrieved by using remotely sensed data. The SM was successfully estimated from several meteorological elements and remote sensing information. Kahle [34] has also conducted many studies on TI models, proposing different approaches for solving one-dimensional heat conduction equations; he attempted to integrate remotely sensed data to estimate TI for large regions. In addition, [19] in their study have simplified the latent and sensible heat flux expression combining the surface one-dimensional heat conduction equation with the energy balance principle by using Fourier transformation to introduce a comprehensive surface parameter (B) as a function of soil emissivity, air humidity, temperature, and other meteorological factors. Because of the requirements of a large number of observed ground data in the B solving process, this approach is unavailable for many regions. Many TI models have treated thermal soil characteristics as a simplified linear function of temperature [34,35]. However, [36] in their study considered that this relationship is nonlinear and includes factors such as the effects of night frost [29]. A simple proxy of TI is the apparent thermal inertia (ATI), which can be derived from multispectral remote sensing imagery by measuring the spectral surface albedo and diurnal temperature variations [32].

In this work, we propose a simplified approach based on the integration of in situ measurements with reflectance and thermal remote sensing data to extend the SM information (derived from the in situ measurements) to a wide area characterized by bare soils and low vegetated agricultural fields. To this aim, a calibration procedure was applied to a high-spatial-resolution (2 m) airborne and satellite remotely sensed dataset.

The following sections describe the materials and methods, the study area (Section 2.1), the remote sensing and field data (Section 2.2, Section 2.3 and Section 2.4) used for this study and the Thermal Inertia (TI) and SM estimation methods (Section 2.5). In Section 2.6, a comparison of the SM map with an available landslide catalog map of the area is presented. In Section 3, the results and discussions are presented and evaluated. Finally, conclusions and future perspectives presented based on our results.

## 2. Materials and Methods

### 2.1. Study Area

**Figure 1:**
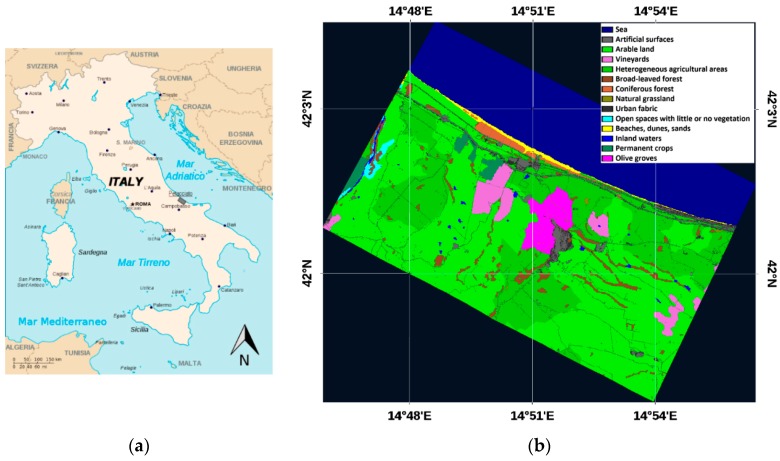
(**a**) Location of the study area. The black box indicates the TASI-600 and WV2 acquired area. (**b**) Land cover classification of the study area up to the third level of the Corine Land Cover 2012.

The study area used for the execution of the field and airborne surveys includes the northern portion of the Municipality of Petacciato (Molise, Italy; Figure 1a), downstream from the town to the sea and covering a total area of about 65 km^2^. In the coastal section of the Petacciato study area, which is affected by the periodic reactivation of extensive landslides [36], outcrops a marine blue clay sequence from middle to lower Pleistocene in age, characterized by 10-cm thick layers, generally recognizable by the presence of thin silty-sandy levels [37]. From the climatic point of view, the territory is part of the Mediterranean region, the thermotype is meso-Mediterranean—termo-Mediterranean and the ombrotype is dry/humid–sub-humid [38].

The land use consists of predominantly arable land (53%) and heterogeneous agricultural areas (25%), interspersed with olive groves and vineyards (7%) and sporadic arboreal patches of original Mediterranean vegetation (5%). The main kind of crops occurring in the study area at the TASI acquisition day was wheat. The countryside is dotted with rare rural houses that, in recent times, have been gradually increasing in number, especially near the inhabited centers, giving rise to a sort of spontaneous aggregation that creates a inorganic and disjointed filamentous in the territory.

Figure 1b shows the detailed land cover map of the study area with the land cover classification up to the third level of the Corine Land Cover 2012 [39].

### 2.2. TASI Thermal Airborne Data

On 12 April 2018, two flight campaigns were carried out in the same day on the Petacciato area with a pushbroom hyperspectral thermal sensor (TASI-600, ITRES, Calgary, AB, Canada) installed on-board a P68 Partenavia airborne. The sensor has a 40° field of view and operates in the spectral region of the long wave infrared (LWIR) with 32 bands of 109.5 nm amplitude for a continuous spectral coverage in the wavelengths between 8.0 and 11.5 μm [40].

The first airborne survey was carried out on 12 April in the early morning between 06:38 and 8:08 UTC to estimate the temperature of the soil with the absence of solar load, while the second one was operated in the same day during daytime, between 10:50 and 12:08 UTC, in order to evaluate the daily increase in surface soil temperature.

A total of 24 TASI images were acquired and processed using radiometric and geometric correction procedures partly provided by the construction company (ITRES Research Limited, Calgary, Alberta, Canada) and partly developed by the CNR IMAA researchers [41].

The techniques used to correct TASI 600 airborne images from atmospheric effects and for the separation of temperature and emissivity are described below.

Thermal images are affected by atmospheric attenuation, diffusion, and emission phenomena, as a function of the acquisition height and atmospheric conditions at the acquisition time. The compensation of these effects is an important issue to quantitatively retrieve parameters as brightness temperature, kinetic temperature, and emissivity. Even in the bands corresponding to relatively transparent atmospheric windows (3–5 μm and 8–12 μm), there is a sufficient amount of interference from the atmosphere, which results in significant errors in the estimation of these parameters. Literature studies [42] show how atmospheric compensation algorithms, based exclusively on the data acquired on the scene by the thermal sensor such as the in-scene atmospheric compensation (ISAC) method, offer an accurate method to compensate for atmospheric effects in the 8–12 µm spectral range.

The ISAC algorithm, described by [42,43], was applied for the correction of thermal airborne data by [44,45,46] and used for the TASI-600 imagery. The ISAC algorithm assumes that some of the pixels in the scene behave like a black body without the need of knowing the exact position or temperatures values (‘max hits’ method). For each wavelength, the measured radiance values are compared with those derived from the reference ‘black body’, and the results of the comparison are reported in a scatterplot. The parameters for the correction are directly extracted from the interpolation of the ‘max hit’ pixels (where the emissivity is considered close to 1) of the scatterplot through a linear regression.

The TES method, developed and evaluated by [47,48,49,50,51], was first used to obtain the standard products of ground temperature and emissivity for the ASTER thermal sensor (Advanced Spaceborne Thermal Emission and Reflection Radiometer). This algorithm allows to determine the absolute value of the spectral emissivity using the measurements made in N (at least 4) spectral bands in the TIR range.

In this algorithm, for each pixel, the temperature is calculated on all the spectral channels using a constant emissivity value, usually set at 0.96 [51]. The highest temperature value, among all the bands, is considered the temperature value of the pixel. For this study, we applied the TES algorithm as implemented in the ENVI 5.1 software (HARRIS Geospatial Solutions, Broomfield, CO, USA) to retrieve from the TASI-600 at-ground radiance images the temperature images.

**Figure 2:**
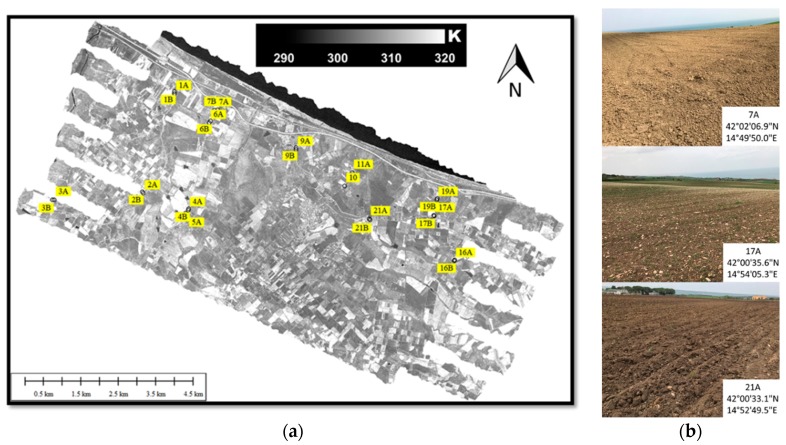
(**a**) TASI-600 temperature images acquired on 12 April 2018 at midday over the Petacciato study area (center coordinates: 42°00’ N; 14°51’ E), with overlapped the soil sampling points (sampling points label is indicated in the yellow boxes only for visualization purposes). (**b**) Examples of three bare/scarcely vegetated soils of agricultural fields of the study area from which the soil samples (7A, 17A, 21A) were collected for the SM and texture laboratory analysis.

Figure 2a shows as an example the TASI-600 midday flights temperature images for the Petacciato study area, with overlapped soil sampling points used for calibration and validation purposes. In Figure 2b an example of three bare/scarcely vegetated soils of agricultural fields of the study area from which bare/scarcely vegetated soil samples (7A, 17A, 21A) were collected and further used for the SM and texture laboratory analysis.

### 2.3. WorldView 2 Satellite Data

The World-View-2 (WV2) imagery used for this study to estimate the albedo of the study area was acquired on 18 April, 2018 at 10:06 UTC. The WV2 satellite sensor was launched on October 2009 and it acquires, from an altitude of 770 km, multispectral images with eight bands in the VNIR spectral range (0.400–1.040 µm), with a ground sampling distance (GSD) of 2 m. The imagery was provided in radiance values by DigitalGlobe (DigitalGlobe, Inc., Westminster, CO 80234, USA) through the Italian reseller Planetek Italia (Bari, Italy) and, then, it was ortho-rectified using the provided rational polynomial coefficients (RPC) coefficients and LiDAR data.

Concerning the orthorectification of WV2 imagery, it refers to the pixel-by-pixel image correction that compensates the topographic distortion and the effect induced by the presence of orographic reliefs within the acquired scene. This procedure requires the knowledge of the digital model of the terrain and a series of information related to the acquisition geometry and dynamic. The LiDAR data provided by the National Geoportal of the Ministry of the Environment and Protection of Territory and Sea (MATTM) were used. The LiDAR data were elaborated with the ENVI LiDAR software to obtain a DTM that covers the whole area of interest at a spatial resolution of 1 m. In addition, the RPC coefficients supplied as ancillary data of the WV2 image were used in the ENVI orthorectification tool.

Then, WV2 orthorectified radiance imagery was atmospherically corrected. For this purpose, numerical models (also known as radiative transfer codes) are used to simulate the behavior of the radiation in the atmosphere, defining the absorption of gases and modeling the scattering processes. These models require inputs on weather conditions, such as atmospheric gas content, humidity, visibility, etc. Among the best-known numerical models, there are LOWTRAN, MODTRAN, and 6S [49].

The atmospheric correction was carried out using a software developed in the IDL (HARRIS Geospatial Solutions, Broomfield, CO, USA) language at the IMAA CNR (Italy), which implements the model

(1) $$L=\frac{A\rho }{1-\rho_{e}S}+\frac{B\rho_{e}}{1-\rho_{e}S}+L_{a}$$

where, *L* is the at-sensor radiance, $L_{a}$ is the atmospheric contribution, *S* is the spherical albedo of the atmosphere, *A* and *B* are quantities that depend exclusively on the atmospheric characteristics, *ρ* and *ρ_e_* are the pixel reflectance and the average reflection of one of its surroundings respectively.

**Figure 3:**
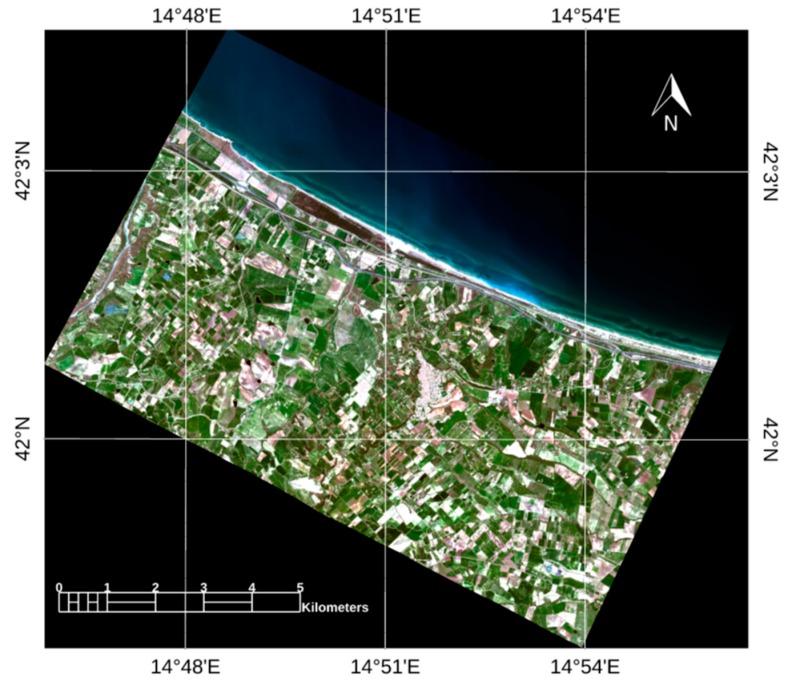
Corrected to reflectance WV2 imagery clipped to the TASI flyover area. RGB (R: 659 nm; G: 546 nm; B: 478 nm) imagery.

After launching a series of MODTRAN simulations for the estimation of the radiative quantities (*A*, *B*, *S*, *L*, *L_path_*), the software needs to operate a couple of correction run to retrieve *ρ*. In fact, to estimate *ρ*, *ρ_e_* should be estimated first and, therefore, *ρ* is required to estimate *ρ_e_*. To this aim, a first correction run is applied considering *ρ_e =_ ρ* in Equation (1). Then, the obtained approximated ρ is averaged to estimate *ρ_e_* using a point spread function as in [49]. Finally, in the second run, Equation (1) is inverted to obtain *ρ*. Figure 3 shows the WV2 imagery corrected to reflectance.

Finally, the albedo was estimated using the equation

(2) $$a=\frac{\sum_{n=1}^{N}\rho_{n}I_{n}}{\sum_{n=1}^{N}I_{n}}$$

where, *N* is the number of bands, *n* is the band index, $\rho_{n}$ is the spectral reflectance at band *n*, and $I_{n}$ is the incident spectral irradiance convolved with the instrument spectral response function of band *n.*

### 2.4. Field Data

Simultaneously with the acquisitions of the TASI thermal images (12 April 2018), a ground campaign was carried out to collect soil samples to be used in the calibration procedures and in the validation of the SM maps. The samples were analyzed in the laboratories of the CNR-IMAA to measure the soil water content and texture.

The sampling of the plowed agricultural land was carried out on different fields with bare soil (i.e., without vegetation). In particular, 27 samples of sub-superficial sediment were collected all at the same depth of about 10 cm according to the scheme illustrated in Figure 2. The sampling of the soil samples was followed by immediate sealing in appropriate containers, which were stored at +4°C until analysis (within 24 h).

For the characterization of the soil in the natural state, we proceeded with the determination of some properties (indices) which included the water content and the granulometry.

The water content (W) is defined as the percentage of water present in the soil with respect to the solid phase. To determine W, each soil sample was placed in a container of known weight *P* and weighed obtaining the measure *P*1. After having placed the container with the sample in a ventilated oven at 105 °C for 2 days, the measure was repeated obtaining *P*2. The value of W was, thus, derived through the formula

(3) $$W=\frac{P1-P2}{P2-P}\times 100$$

The granulometry was estimated by means of two techniques: washing and sieving. The washing of the material in the 0.075 mm sieve allowed us to obtain weight information on the silty fraction joined to the clay fraction present in the sample; while with the dry sieving the gravel fraction was separated from the sandy one. Before proceeding with the washing, the sample was immersed in water and disaggregated with the aid of mechanical shovel agitators, which was left running for one night. Subsequently, the sample was washed in a sieve with a 0.075 mm diameter mesh, taking care to protect it with one having a 2 mm mesh. For sieving, the sieve n. 10 of the ASTM series was used with a height of 80 mm and a diameter equivalent to a 2 mm mesh and a bottom collector. The sediment fraction of diameter greater than 2 mm accumulates on the sieve, separating the gravel fraction >2 mm from the sandy one <2 mm. Subsequently, the weight of each single fraction retained within the sieve and the bottom collector was estimated, evaluating the percentage with respect to the total initial weight for the two different granulometric classes to which they belong.

### 2.5. Thermal Inertia and Soil Moisture

**Figure 4:**
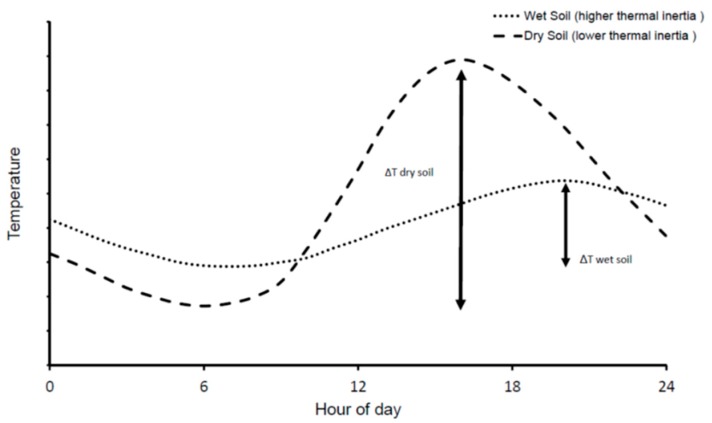
Comparison between the daily trend of water and dry soil temperature (modified from [54]).

The Earth’s surface has thermal characteristics and different daytime temperature trends, according to which it is composed. The daily temperature trend illustrates how fast an object heats up and cools during a day [52]. Figure 4 illustrates an example of the daily pattern of water temperature and dry soil. The differences in daily trends derive from the different thermal properties of the two materials [52]. A parameter that describes the thermal behavior is TI, which is defined as the resistance of a material to heating. The TI depends on three factors: the thermal capacity *c* (the energy needed to increase the temperature of a material of 1 Kelvin per unit of mass), the density *p* of the material and its thermal conductivity *k* [53]

(4) $$TI=\sqrt{c*p*k}$$

Variations in *TI* lead to changes in *ΔT* [53], which is the difference between the maximum and minimum temperature that occurs during a diurnal solar cycle (Figure 4). The relationship between *TI* and SM can be determined quantitatively from the changes in soil temperature. Low *TI* indicates a low resistance to temperature change, resulting in a high *ΔT* (e.g., dry soils). The opposite happens to surfaces characterized by high *TI* (e.g., wet soils).

After the temperature map retrieval, the ATI equation (Equation (5)) was calculated. As shown in the study of [32], the ATI could be used as a simple proxy of the TI by using as input spectral surface albedo and the diurnal temperature variations. Van Doninck et al. [55] claim that the diurnal temperature amplitude should be derived using two or more temperature measurements as only one thermal imagery results would have low accuracy in the ATI estimation. In our case, having an aerial platform available, we were able to optimize the ATI assessment with two thermal acquisitions.

It is useful to remark that the proposed method has an assumption—i.e., that the soil diurnal temperature increase is mainly due to the solar irradiation—and, therefore, in order to apply the proposed method the solar illumination conditions have to be homogeneous in the whole area and between the two thermal images acquisitions. For this study, we have planned the thermal imagery acquisition on the base of meteorological forecast and therefore both images were acquired in clear sky conditions.

As well as remote sensing does not allow the direct derivation of *TI* (*c*, *p*, and *k* can only be measured in situ), an estimate can be obtained with the so-called ATI [16], which according to [30] can be represented by the equation

(5) $$ATI=c\frac{1-a}{\Delta T}$$

where, *a* is the albedo of the single pixel in the VNIR band and *c* is a constant term that we set to 1 including it in the parameters assessed by the calibration procedure for the SM assessment described later on in this chapter. However, as the *c* value depends on different parameters and conditions (e.g., the sky illumination conditions, the interval between the two thermal acquisitions), it has to be calculated every time that the method is applied in order to calibrate the TI value. The term (*1 − a*) compensates for differences in temperature due to the reflectance of the soil rather than its thermal characteristics.

In this study, the ATI map was derived using the airborne TASI-600 (for the diurnal ΔT estimation) and satellite WV2 (for the albedo characterization) images.

In detail, as the relationship between ATI and SM shows good dependence only in the presence of poorly or scarcely vegetated soils [21], the WV2 image was used to create a mask of bare/scarcely vegetated soils using the most commonly used vegetation index for this purpose, the normalized difference vegetation index (NDVI) [22]. The WV2 bands used for the NDVI calculation are band 5 (658 nm) and band 7 (832 nm). The value of this index ranges from −1 to 1. The common range for green vegetation is 0.2–0.8 [23]. The NDVI threshold applied for the WV2 imagery is 0.25, which is a threshold that can separate green vegetation from bare/scarcely vegetated soils fields in the selected study area. The resulting percentage of bare soil compared to cultivated area, obtained from merging the arable land and heterogeneous agricultural areas classes (Figure 1b), is of 35%.

In order to convert ATI values into SM, a calibration procedure has to be carried out. Although a non-linear relationship has proved more appropriate to represent the relationship between ATI and SM [56], in our case, a slight advantage in the use of a linear relation was observed. As we are mainly focused on the integration of in situ and remote sensed data, rather than on the best assessment of the correlation formula, a linear regression model was carried out using the SM values calculated for the in situ collected samples and the corresponding ATI values.

The accuracy of the models was evaluated observing the root mean square error (RMSE) of cross-validation using 13 folds—i.e., a leave-two-out cross validation—which is widely used when the number of available samples for calibration does not allow to split the dataset into calibration and validation [57]. After the validation process, the general linear regression model was applied to all the bare and poorly vegetated soils in order to obtain the SM map.

### 2.6. SM and Landslides

**Figure 5:**
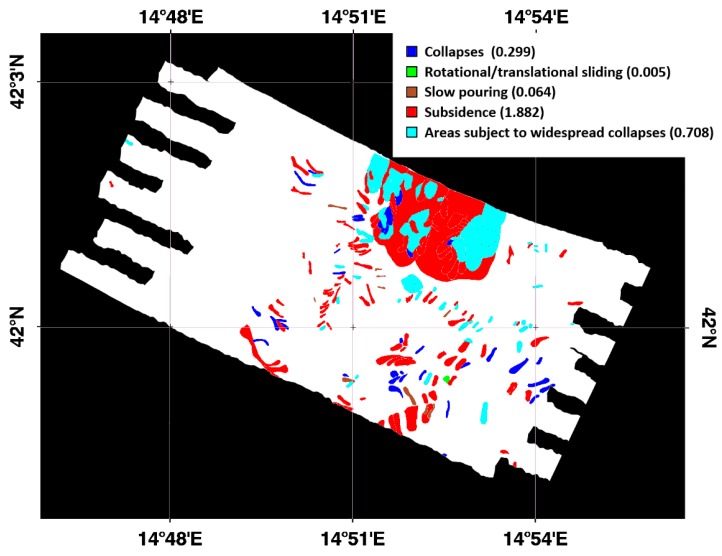
Landslide types occurring in the study area from the landslide catalog [58], in the legend the colors represent the different landslide types and the area extension is expressed in km^2^.

Although the focus of the study is to estimate the surface SM, in the framework of the founding project (see acknowledgments), a comparison between the SM and the landslides’ catalog obtained from the Italian National Geoportal of the Ministry of the Environment and the Protection of Land and Sea [58] was also carried out. The Italian Ministry of the Environment in collaboration with the Regions set up the landslide catalog on the bases of a historical database, in order to provide a complete and updated picture of the distribution and types of landslides occurring in Italy. The catalog is organized in information layers according to the geometries and the types of movement and discriminates between the following landslide types: collapse, rotational/translational sliding, expansion, slow pouring, rapid pouring, sinking, complex, deep gravitational slope deformations, areas subject to widespread collapses/reversals, areas subject to widespread collapses, and areas subject to widespread shallow slopes. Figure 5 shows the landslide types occurring in the study area as derived from the landslide catalog [58]. In the legend of Figure 5, the different colors depict the landslide types and the area extension is expressed in km^2^ in the round brackets.

Furthermore, to figure out if, as described in [59,60], a relation between the SM map and the landslides interesting the study area exists, a statistical analysis was carried out. The SM frequency distribution was computed on the SM map for each sliding type of the catalog occurring in the area and for the background (pixels outside the sliding areas). The separability between the frequency distribution of the background pixels $D_{bck}\left(SM\right)$ and of the various sliding types $D_{sl}\left(SM\right)$ was evaluated by means of a separability index from the background ($SI_{bck}$) defined as

(6) $$SI_{bck}=100\times \left(1-\frac{\int D_{sl}\left(SM\right)D_{bck}\left(SM\right)dSM}{\sqrt{\int D_{sl}\left(SM\right)dSM\int D_{bck}\left(SM\right)dSM}}\right)$$

## 3. Results and Discussion

In this study, we have used high-resolution airborne thermal imagery (TASI-600, 1 m of GSD) coupled with reflectance high-resolution satellite data (WV2, 2 m of GSD) to estimate SM map over an extensive agricultural area in central Italy. It is important to remark that we could not directly connect surface temperature variations with the surface SM, but only by integrating the surface TI variations on bare and low vegetated soils with spectral surface albedo and ground SM measurements, it is possible to estimate SM over them. The limitation of the proposed method is that the calibration procedure used to obtain SM data from TI must be repeated each time the procedure is applied. This because the calibration coefficients for the SM retrieval are influenced by different factors, e.g., solar illumination conditions and thermal image acquisition interval.

The temperature, ATI, and SM maps are presented and discussed in the following sections.

### 3.1. Temperature and ATI Maps

**Figure 6:**
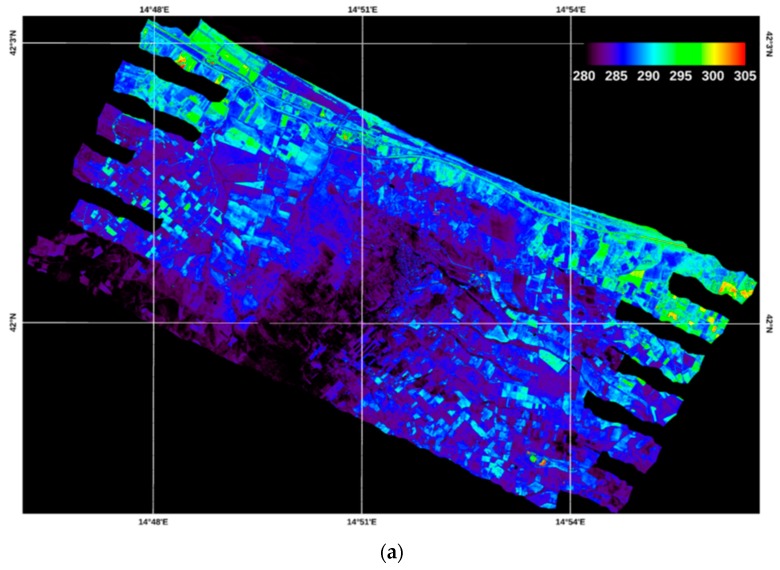

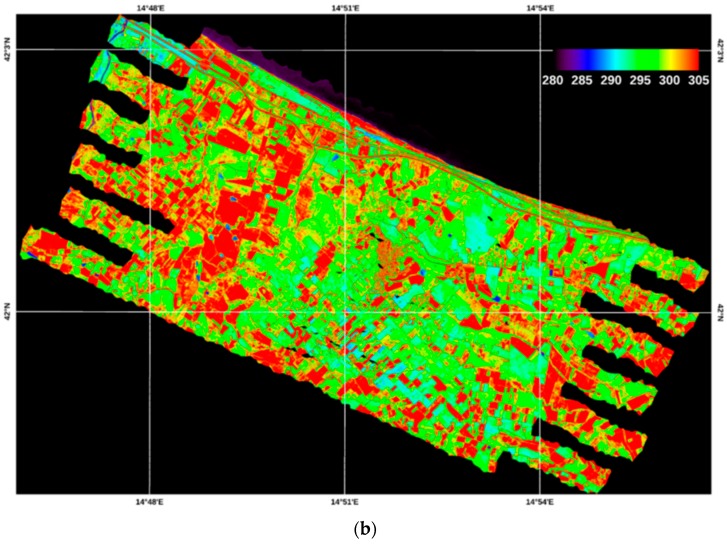
Ground temperatures obtained from the TASI images in the morning (**a**) and during maximum solar load (**b**).

By applying the TES algorithm to the atmospherically corrected TASI-600 airborne images, two temperature maps were obtained. Figure 6 shows the retrieved temperature maps. In particular, Figure 6a shows the temperature map of the morning flights, ranging from 270 to 320 K; while, Figure 6b those of the early afternoon, which vary between 280 and 330 K. This temperature range is in accordance with the sensitivity studies conducted by [61], which show that the surface moisture impacts much more on the temperature than on albedo variations and that, for an accurate estimation of SM, it is sufficient a temperature variation of the order of 10 K.

**Figure 7:**
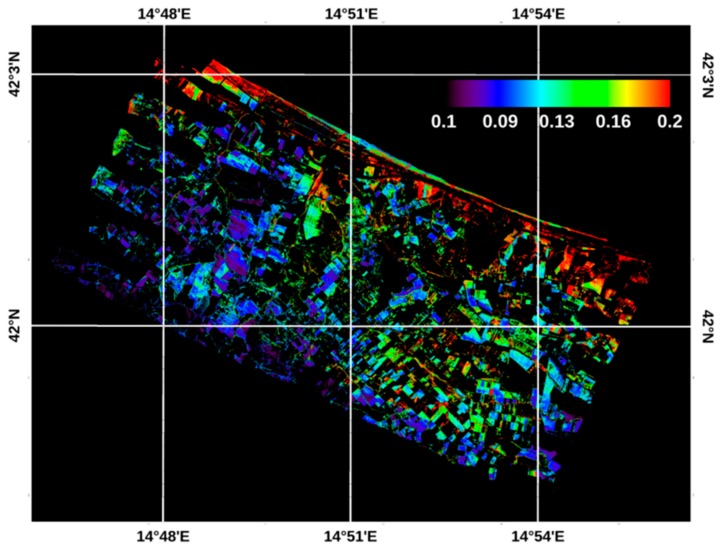
ATI map of the poorly vegetated and bare soils of the study area. The ATI values in the legend are expressed in K^−1^ unit.

Figure 7 shows the map of ATI obtained for bare and poorly vegetated soils, with the relative legend. The ATI map was obtained by using the two TASI images, applied for the *ΔT* retrieval, and the WV2 image for the albedo estimation. Compared to the temperature images, the ATI map shows a strong, non-ambiguous dependence on the morphology occurring in the study area, which is characterized by drier inland hill areas and more humid flat coastal areas. In fact, the ATI map values range from 0.07 to 0.22, with the highest values distributed in the fields next to the coastal areas. In these areas close to the sea, the ATI values consistently exceed the range related to the field sampling points, thus lowering the accuracy of the method for them.

### 3.2. Soil Moisture Map

The laboratory analyses’ results on the collected soil samples (i.e., 27 samples of the first 10 cm of soil collected in different sites of the study area), the percentage of their different granulometric fractions, and the water content of each individual sample, are shown in Table 1. Looking at the laboratory results for the soil samples texture, we can notice that they are mainly composed of silt/clay fraction, with the only exception of the sample 17A, showing a high gravel percentage (23.1%) and of samples 1B and 11A (collected on bare soils near the coastal area) with a sand content of 31.98% and 49.8%, respectively. This is in accordance with the work of [37] that argue that the coastal section of the Petacciato study area is characterized by 10-cm thick layers with thin silty-sandy levels. Samples 5A and 19B show the highest silt/clay content with 96.5% and 96.7%, respectively.

The laboratory analysis highlighted that all of our samples have a water content (defined as the percentage of water present in the soil compared to the solid phase) varying from 7% to 13% and that they are almost homogeneous in terms of texture (the silt/clay fraction prevails with values up to 96%). This indicates that the observed changes in diurnal thermal values are principally due to surface SM changes, as confirmed by [62,63,64].

**Figure 8:**
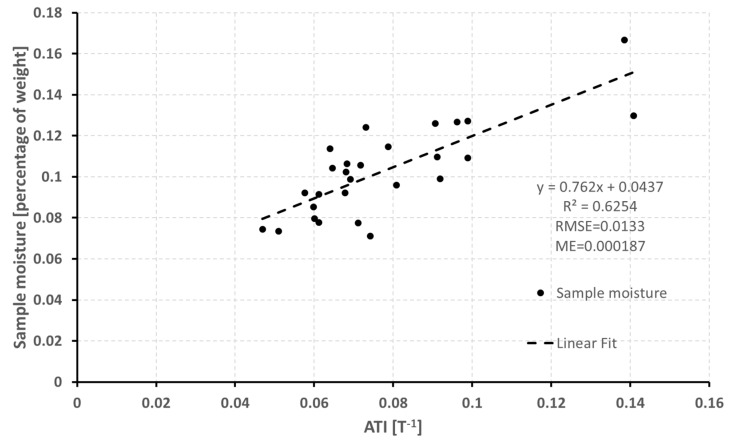
SM vs. TASI ATI values. The dashed line represents the linear fit with slope 0.762 and intercept 0.0437.

In order to convert the retrieved ATI values into SM, a calibration procedure was carried out using the laboratory SM values of the collected samples. The general linear regression model between the ATI retrieved values and the SM values is shown in Figure 8. The RMSE of the cross-validation is 0.0133 with a standard deviation of 0.005, which is slightly higher than the RMSE of the general model (0.013). The mean error (ME) is 0.000187.

Concerning the ATI model applied for this work (Equation (5)), previous studies have shown that it is accepted [29], and extensive studies were conducted in different areas with different SM levels. In [29], the authors demonstrate that TI is highly correlated with SM and that the higher the TI, the less is the variation of T. As verified in our study (see results shown in Figure 8) and in [29], there is a good relationship between TI and SM, and, therefore, it is possible to quantitatively determine the SM from the diurnal changes in soil temperature. Moreover, [65] in their study combined an ATI model using MODIS data and quantitatively estimated the SM and sediment availability for a white ShashaQiu field in New Mexico. The authors used two constants (N and C) for the ATI calculation, taking into account for variations in solar flux with latitude and solar declination, whereas in our study we used a simplified model with only one constant, which strictly depends on the local conditions at the acquisition moment. In conclusion, our SM estimation requires a calibration procedure each time the method is applied. Veroustraete et al. [66], in their study, applied the ATI approach to retrieve SM in soils using MODIS satellite data in China. They found a R^2^ value of 0.8 between ATI and ground SM measured with time domain reflectometry (TDR). The R^2^ value is in fully accordance with our results (see Figure 8), although the method used for measuring the ground SM are different. In the study of [67], to accurately derive the SM, the authors monitored the spring drought by using the improved ATI model for the Hebei Plain. The authors used the Chinese Huan Jing environmental satellite (HJ-1) to estimate the drought in the vegetated mixing zone. They concluded that the traditional ATI model was the most suitable for bare land areas and that the inversion accuracy of the mixed pixels was poor.

In view of the results obtained by [67] and because our simplified ATI model works for bare soils and low vegetated areas, we applied a mask of the vegetated areas on the ATI map before calibrating it with the coefficients obtained from Figure 8. Beside the vegetated areas, the artificial surfaces, urban fabric, and inland waters were masked. The artificial surfaces and the urban fabric were masked because it is not possible to measure the SM under these kinds of manmade surfaces; while, as regard the inland waters, the SM is completely out of the calibration range of the model. Therefore, in this study, only the bare and low vegetation conditions were considered appropriate to derive the SM map.

**Figure 9:**
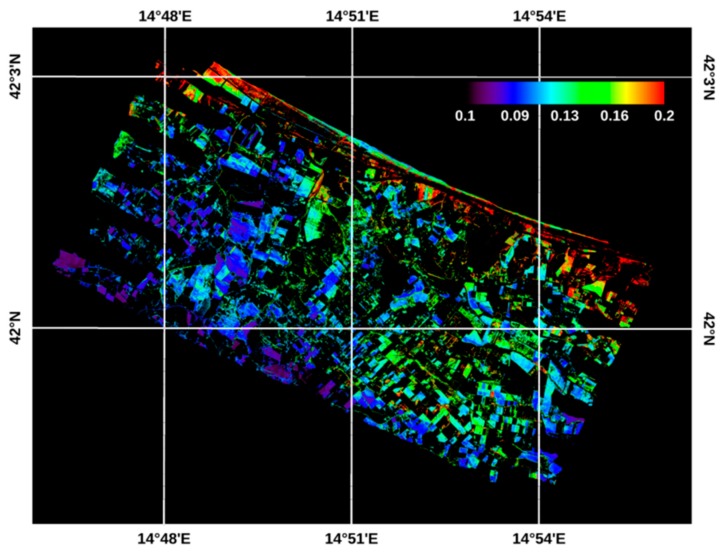
Map of SM obtained from the calibration of the ATI map, the values of the legend are expressed as a percentage of weight (range 0.05–0.25).

Figure 9 shows the SM map obtained for the bare and low vegetated soils of the study area. This figure clearly shows that the lowest SM content has been identified in the southwestern side of the acquired imagery. As for the ATI map, even the SM map shows high values along the coastal areas that are characterized by a high occurrence of thin silty-sandy soils in the upper 10 cm. The retrieved SM values for these areas correspond to the upper SM range limit. Due to the loss of soil samples corresponding to such high level of SM, the results for these areas correspond to a higher uncertainty compared to the other sampling areas. This effect would be reduced if, in the SM calibration procedure, a logarithmic relation had found the best, as in the work of [56]. Nevertheless, with the ground data at disposal, our retrieved linear relation provides the best fit.

**Figure 10:**
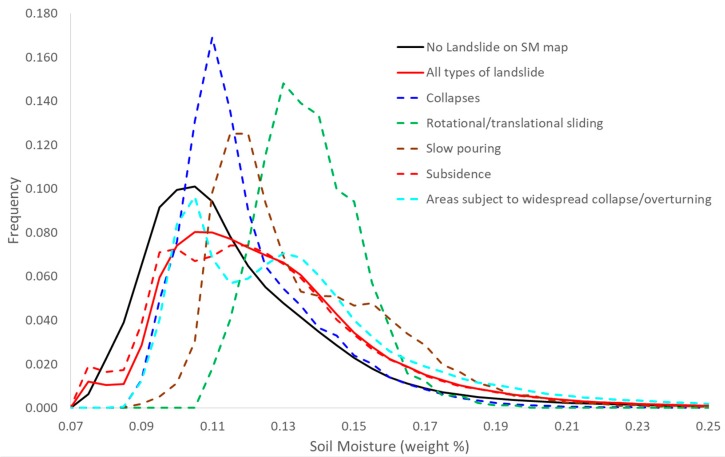
SM frequency distribution related to the background and to the various sliding types.

In addition, as shown on Figure 10, the SM distributions related to the sliding areas present higher values compared to the SM distribution computed on the background. The *SI_bck_* shows different values for the various sliding types as presented on Table 2, ranging from 4.8% of the subsidence landslide type to the 50.6% of the rotational/translational sliding.

Although preliminary, these results seem to be in accordance with the study of [59,60], in which three study regions had slope movements when SM was high and rainfall occurred clearly indicating a relationship among landslide events, remotely sensed retrieved SM, and rainfall data.

## 4. Conclusions

In this study, we present a first attempt to SM mapping for bare and scarcely vegetated soils by integrating high spatial resolution (2 m of GSD) airborne thermal and optical satellite remote sensing data. We have demonstrated, with our test site in Italy, that the surface SM changes influence the diurnal thermal behavior of soil surface temperature and that SM can be retrieved from the calibration of the ATI. The proposed approach is based on the integration of in situ and remote sensing data and uses consolidated literature TI models. For this purpose, ground soil sampling was carried out in different fields in the study area in order to determine the topsoil moisture content and the granulometric composition of the soil samples. These data were used to calibrate the ATI maps obtained on the bases of two different airborne thermal TASI-600 surveys over the study area and using a WV2 multispectral satellite image that was used for the albedo calculation.

We have found that a linear relation exists between the ATI estimated values and the SM of bare and scarcely vegetated soils. The results obtained by means of a linear calibration procedure are satisfactory, with the RMSE of the cross-validation of 0.0133 and standard deviation of 0.005, which is slightly higher than the RMSE of the general model (0.0130). For very wet soil conditions, as in the east side of the coastal area of the study area, the fit of the linear relation of the retrieved model could be improved using a logarithmic relation in the SM calibration procedure.

The main limitation of the proposed method is that the applied calibration procedure must be repeated each time the procedure is applied due to the different solar illumination conditions and thermal image acquisition intervals. Furthermore, if we translate to our results what is known in the literature about surface SM behavior estimation at coarse spatial resolution, we can achieve—at the within field level—an accurate estimate of SM using the actual remote sensing technology is still a difficult and expensive task. This is almost due to SM spatial–temporal variability and to the scale problems characteristic of this application.

From these results, further research will focus on: (i) increasing airborne thermal acquisitions during daytime in order to better define the ATI and to assess its impact on the quality of remotely estimated SM maps; (ii) increasing the ground sampling of surface moisture for bare and vegetated soils in order to the extend and validate the proposed simplified approach to a wide area; (iii) evaluating new models for the estimation of SM in vegetated areas through thermal and reflectance data; (iv) considering other soil properties (e.g., soil texture, structure or type, soil organic carbon) in order to understand their influence on soil surface thermal properties and on the albedo estimation.

## Figures and Tables

**Table 1:** Granulometric results of soil samples collected at Petacciato on 12 April 2018 and analyzed at the CNR IMAA laboratory in Potenza.

Latitude	Longitude	Sample Name	Weight Percentage Variation (%)	Gravel (%)	Sand (%)	Silt/Clay (%)
42.039200	14.817500	1A	10.23%	1.28	4.74	93.97
42.039736	14.817545	1B	9.22%	4.62	31.98	63.38
42.015267	14.807606	2A	7.96%	0.025	5.75	94.21
42.015567	14.807206	2B	9.21%	0.12	6.18	93.68
42.013574	14.778780	3A	7.35%	0.012	5.84	94.14
42.013658	14.778207	3B	7.45%	0.1	9.6	90.29
42.011272	14.822114	4A	7.76%	0.82	6.42	92.75
42.011556	14.822225	4B	9.88%	0.29	6.247	93.45
42.010208	14.821804	5A	8.54%	0.29	3.2	96.5
42.010508	14.821264	5B	7.77%	0.78	6.15	93.06
42.032228	14.828806	6A	9.15%	0.19	18.69	81.11
42.032639	14.829167	6B	10.64%	0.23	10.26	89.5
42.035236	14.830550	7A	10.42%	0.19	13.28	86.52
42.035400	14.831486	7B	11.38%	0.38	14.06	85.54
42.025833	14.856911	9A	10.57%	1.8	14.15	84.03
42.026250	14.856839	9B	12.41%	0.66	10.74	88.58
42.017053	14.872550	10	12.68%	6.42	3.52	90.06
42.020264	14.875036	11A	10.92%	4.23	49.8	45.96
42.019786	14.874883	11B	12.59%	0.87	4.4	94.7
41.999111	14.907997	16A	9.60%	1.44	7.62	90.94
41.999294	14.908156	16B	7.12%	2.8	5.98	91.21
42.009892	14.901481	17A	9.90%	23.1	17.9	58.9
42.010067	14.901300	17B	12.72%	8.4	17.4	74.2
42.013753	14.902258	19A	10.98%	1.68	5.33	92.98
42.014103	14.902478	19B	11.47%	2.96	2.96	96.7
42.009194	14.880428	21A	16.68%	2.53	19.08	78.37
42.008972	14.880714	21B	12.98%	2.21	15.9	81.86

**Table 2:** *SI_bck_* computed for the various landslide types

Landslide type	*SI_bck_* (%)
All types of landslides	5.2
Collapses	9.7
Rotational/translational sliding	50.6
Slow pouring	27.9
Subsidence	4.8
Areas subject to widespread collapses	10.3
